# A Feminist Ethic of Care for the Veterinary Profession

**DOI:** 10.3389/fvets.2022.795628

**Published:** 2022-03-17

**Authors:** Vanessa Ashall

**Affiliations:** Science and Technology Studies Unit (SATSU), Department of Sociology, University of York, York, United Kingdom

**Keywords:** emotional sponge, ethnography, auto ethnography, euthanasia, emotions, relationships, lived experience, moral stress

## Abstract

*I can still see the dog's face as its eyes connected with mine, framed by the black bin bag it had been carried in. I can still hear the clicking sound, louder than the animal's shrill cries, made by a mass of maggots moving against one another beneath the dogs matted fur, moistened by fluids leaking from its damaged flesh. My hands were shaking with panic and rage and I could hardly draw up the euthatal into the syringe quickly enough. I wanted to put an end to this, immediately. As the lethal fluid flowed into the tiny vein the dog's body finally relaxed. At my hand, like so many others, she had ceased to exist. Through the window I could see her owners waiting outside in the sunshine to pay me and I thought about the silky feel of the fur which covered an expensively shaped head. I knew this dog was loved once*.

This paper develops two neglected areas of veterinary thought; anthropological studies of the veterinary profession and feminist care approaches in veterinary ethics. I argue that the development of veterinary anthropology is crucial to advancing our understanding of veterinary lived experiences, through highlighting the previously under acknowledged emotional, relational and contextual realities of veterinary practice. I further propose that an ethic of care for the veterinary profession, which meaningfully connects with veterinary lived experiences, may provide a valuable approach through which to further develop veterinary ethical thinking. I share an autoethnographic account of a difficult veterinary encounter, which I then analyse using a novel feminist care approach. Through analyses centered on both emotional and relational aspects of veterinary care, I challenge the boundaries of traditional veterinary ethical approaches in terms of the scope, scale and complexity of veterinary ethical decision making. I describe the concept of emotional sponge work in veterinary practice and outline its potential impact for advancing understanding of both veterinary well-being and the profession's societal role. Finally, I propose that a feminist ethic of care might provide a framework for redefining the focus of veterinary professional responsibility, beyond animal health and toward the maintenance of healthy relationships between humans and animals.

## Introduction

Whilst working as a newly qualified veterinary surgeon I dealt with a disturbing case which has stayed with me for over 20 years. It was not, unfortunately, the only sad and shocking case that I have seen in my career. Rather it is one of many such stories, the like of which all vets could tell. I share it here in order to illustrate the value of developing anthropological understandings of veterinary medicine, specifically in relation to developing the field of veterinary ethics. I argue that developing such stories of veterinary medicine may help us to reframe the ways in which we think about veterinary ethics and make the case for a development of feminist care approaches.

This paper has two separate but connecting aims. I firstly introduce anthropology and ethnographic methods as valuable in further developing our understanding of the veterinary profession and the lived experiences of being a vet. I secondly introduce feminist ethical analysis as an underexplored area in veterinary ethical debate, and show how such an approach brings to light previously neglected aspects of veterinary ethical decision making.

In the methods section of this paper, I define the field of anthropology and describe the ethnographic methods upon which it is based. Through a scoping review of anthropological and ethnographic literature, I outline the ways in which our understanding of the veterinary profession may be developed through such approaches. I suggest that the unique detail of anthropological studies meaningfully connects lived experience with academic studies of the profession. In the second part of the methods section I go on to demonstrate the limitations of traditional veterinary ethical analytic approaches for the meaningful analysis of such accounts and propose an alternate feminist care approach.

In the results section of the paper, I firstly share novel auto ethnographic data in the form of a veterinary story arising from my own experiences working in the profession. I share in ethnographic detail the real events which surrounded a difficult veterinary euthanasia, and my subsequent interactions with the animal's owners, the RSPCA and my colleagues. I go on to undertake a feminist ethical analysis of this data, through extending two important concepts from existing feminist literature to this novel account of a veterinary encounter. I propose the novel concept of ‘emotional sponge work’ as a useful contribution to the development of a veterinary ethics of care.

In the final discussion section, I explore the significance of my auto ethnographic feminist veterinary analysis for the development of the field of veterinary ethics. I conclude by arguing that a feminist care approach might considerably alter the scope and scale of veterinary ethical decision making. I suggest that the development of such an approach may be critical to advancing our understanding and effective management of veterinary moral stress.

## Materials and Methods

In this methods section, I first introduce the emerging field of veterinary anthropology. I focus on what ethnographic research methods can offer studies of veterinary practice, as an adjunct to more traditional research methods in veterinary science. I then introduce feminist approaches to veterinary ethical analysis, situating them within the context of feminist challenges to traditional ethical understandings of veterinary medicine and of science more broadly.

The aim of this methods section is to show how the ethnographic methods of anthropology and feminist ethical analytic approaches might together be applied to researching and analyzing neglected aspects of veterinary practice.

In the results section of this paper these methodological approaches are applied through the development of a novel autoethnographic account of a historic veterinary encounter and a subsequent feminist ethical analysis of the described scenario.

### Anthropology and (Auto) Ethnography

In this section, I define anthropology and the ethnographic methods upon which the field is based. Through a scoping review of literature which uses ethnographic methods to explore both veterinary and animal laboratory settings, I illustrate some of the ways in which ethnographic methods might be shown to develop our understanding of veterinary lived experiences and the reality of veterinary practice.

#### Anthropology and Ethnographic Methods

I begin this section with an observation on veterinary research methods, which indicates that there is a recent trend toward embracing non-traditional research methods in veterinary science. Most notable is the emergence of qualitative research methods in published veterinary research. For example, the thematic analysis of interview data arising in the veterinary setting (1) and the use of participant observation methods in veterinary practice (2). In this paper I further extend the emerging qualitative trend in veterinary medicine. Through engaging with anthropological literature and ethnographic methods I demonstrate how ethnographic accounts of veterinary medicine can add to our understanding of what vets do and what it means to be a vet.

Anthropology is described as the study of human origins, societies and cultures (3). Contemporary British social anthropology is considered distinct from physical anthropology, which is itself concerned with the study of biological aspects of humanity such as paleontology and genetics (4). In social anthropology the practice of long term participant observation is a standard method, with the aim of gaining a close and intimate familiarity with a given area of study through an intensive involvement with people in their natural setting (4).

The term most commonly linked with the research techniques of social anthropology is ethnography, usually applied to the acts of both observing directly the behavior of a social group and producing a written description (4). As such, the focus and methods of anthropology are undoubtedly different to those traditionally associated with veterinary science. Ethnographic methods bring into focus non-scientific dimensions of veterinary practice and situate them within the complex socio-cultural and political contexts which support and are supported by the profession. This contrasts, perhaps uncomfortably for some, with the scientific methods of knowledge production which have traditionally underpinned veterinary practice, whereby the elimination of contextual or individual influences has long been the hallmark of robust scientific enquiry.

The novel data presented in this paper is auto ethnographic. Applying ethnographic methods to oneself, through auto ethnography, involves the use of personal experience to examine and/or critique cultural experience (5). It is suggested that auto ethnographies differ from other kinds of personal writing though additional characteristics relating to the purpose and impact of the work. Characteristics of auto ethnographic writing include purposeful commentary/critique of cultural practices, contributing to existing research, embracing vulnerability with purpose and creating a reciprocal relationship with audiences in order to compel a response (5). Auto ethnography is described as a research approach which privileges the individual. It is an artistically constructed piece of work that attempts to portray an individual experience in a way that evokes the imagination of the reader, supporting the idea that individual experiences are a legitimate source of research data (6).

#### Anthropology and Veterinary Lived Experience

In this section, I focus on the detail of several anthropological studies in order to demonstrate what this research approach may offer the profession. I show how such an approach connects with my own lived experiences of veterinary professionalism, and will later argue for the role of detailed ethnographic studies of veterinary medicine in reshaping our ideas of veterinary ethics.

As one recent example, Friese and Latimer (7) explored health entanglements between humans and animals through an anthropological study of their interactions in the laboratory. This work demonstrates how ethnographic studies of the practical and professional practices of animal care might also develop our understanding of the well-being of veterinary staff. Friese and Latimer (7) observe the practical aspects of providing care to an aging laboratory animal, at a level of detail which is not usually observed in veterinary literature:

*One at a time, she scruffs each mouse to look at her underside, palpates her stomach to check for any hardness, and then looks at her teeth. Janet watches the mouse move around the cage, and holds the mouse in her hand to see how she comes off to assess strength. If Janet notices anything wrong with a mouse, she takes note and contacts her line manager to decide on the required next steps. (p. 127)*

This ethnographic study connects both practical and emotional aspects of involvement in professionalized animal care:

*Janet continues that it is hard to see a mouse who is starting to die, and who won't quite make it to an experiment. “To have lived in these cages for two or more years, which must be boring for the mice, and then to not quite make it to the study, it is really sad.” (p.127)*

Through showing how stress might be viewed as a relational element, connecting the lives of laboratory animals and humans, this study offers the veterinary profession an example of the place of ethnographic detail in demonstrating multiplicity and complexity in well-being concepts:

*Stress is being enacted in multiple ways within these interspecies relations. The physical body of the mouse is stressed by age and the emotional body of the mouse is stressed by the boredom of living, for a very long time, in a small cage. Both these stressors affected Janet, which, in turn, creates an everyday form of work-place stress. (p.127)*

The formal study and analysis of such complex relationships of well-being is achieved in this study through the detailed and intimate observations typical of ethnographic methods. I will later argue that the study of moral stress in the veterinary profession would benefit from a similar approach.

Friese and Latimer (7) offer a different view of science to that which scientific research itself presents, through detailing unpleasant laboratory procedures which are not commonly discussed. For example:

*Jake gets a plate of worms from the incubator, which keeps the worms at a steady temperature, and takes them to the bench beside his microscope. He notes that he is pleased because the worms have reproduced well in the incubator and on the food he has given them; his care of the worms has worked. Jake tells Latimer that he is going to dissolve the worms in sodium hypochlorite, or bleach, leaving just their eggs. He continues to explain that this is not very nice, bleaching worms to death. (p.128)*

The juxtaposition of practices of care and killing in this account of Jake's work might feel familiar to a veterinary surgeon, as might his account of his career choices:

*Jake: If I'd been told what it takes to do science. Not to be a scientist, but to do the science... Like the time and the frustation, and the... especially working with biology. I think I would have considered it a lot more heavily. I may have made the same decision, but... I don't think I ever.... That was never really impressed upon me as a student, was the doing of the science. Because in school all you do is learn, it's book work, and it's what I loved! (p.129)*

What it takes to “do” laboratory work and the misunderstandings and assumptions which led to Jake's training as a scientist shows what we may achieve through anthropological studies of veterinary careers. What might we learn from such stories of training and working as a veterinary professional?

It is not only vets' relationships with animals which anthropological studies promise to illuminate. Ethnographic methods have also paid attention to interactions between people and everyday materials, whose significance might be explored in the context of veterinary labor. As one example I show here how Hamilton (8) studies the significance of familiar interactions between veterinary surgeons and biological waste. That vets get dirty is well-understood, but the ethnographic detail of such encounters are, again, rarely encountered in veterinary literature:

*Slurry, sludge, blood, pus, mucous and decaying biological tissues – in short the whole panoply of agricultural detritus. During my fieldwork, these became manifestly evident as inescapable elements of the working lives of the vets (p.488)*

What does veterinary anthropology tell us about the significance of this reality? Hamilton (8) observes that “*The vets seemed to wear their muck stains as a badge of honour and professional pride* (p.489)*,”* connecting the potentially opposing concepts of muck and professional prestige. The observation is made that veterinary expertise involves the skilled application of sensory experiences, elevating veterinary “dirty work” to a professional art form:

*In the routine of examination, vets typically make assessments from what can often appear to be a bewildering array of smells, sights and sounds. Smelling the cow's breath, feeling around the mouth, squirting some milk from a teat, smelling or feeling the texture of excreta, squeezing, flicking, listening to and palpating the cow's body are all commonly observed rituals that they perform in the field (p.490)*

Highlighting the particular contextual significance of sensory experiences is one example of how anthropological research might reinvent formal understandings of veterinary professionalism.

That vets' bodies are trained to respond in specific ways to the bodies of animals and their by-products is not a prominent viewpoint in current veterinary literature. Hamilton (8) suggests that “*It is the vet's own body that becomes his most valuable professional instrument on the farm. Here is an astonishing connection between the primeval and the scientific; the unmediated use of the human body as a scientific instrument”* (p.492).

In Hamilton's (8) work, ethnographic research illuminates the detail of *how* specific veterinary knowledge is evidenced. This contributes to a more nuanced understanding of the basis upon which the profession's social standing has arisen, moving beyond only scientific understandings of veterinary professionalism (p.488).

Through connecting ethnographic detail with concepts of professionalism, the role of “muck” in veterinary practice is elevated beyond a mere practical inconvenience. It is this potential for an altered perspective on veterinary labor which makes veterinary anthropology potentially powerful as a tool in developing veterinary ethical reasoning. I will later show how an ethnographic focus on the sensory and material aspects of veterinary work may play a critical role in developing our understanding of ethical veterinary practice.

Veterinary euthanasia, the focus of my own account in this paper, has already been the subject of some anthropological investigation. For example, Morris's (9) detailed observation of vets, animals and their owners brings to life both familiar and unfamiliar aspects of the practice. For example, that euthanasia frequently entails “negotiation” between veterinary professionals and animal owners illuminates the acknowledged complexity of decision making in this area. However, Morris (9) extends the meaning of creating a good death in veterinary medicine beyond only scientific and welfare understandings of the term. The role of the veterinary professional in creating *the appearance* of a good death for the sake of the animal's owner is a less commonly explored aspect of veterinary professionalism:

*Veterinarians often go out of their way to control the appearance of the dead. To create the most pleasing image, an animal's body may be washed, shaved or groomed and its eyes are always carefully closed. Any faeces or urine excreted after death is wiped clean. The body is sometimes covered with a sheet of disposable absorption padding, towel or blanket. The veterinarian may even take the time to sew up an open trauma wound or cover disfiguring injuries (p. 50)*

Whilst this aspect of veterinary care is, again, likely to be very familiar to those working within the profession, ethnographic research brings such secretive practices, which intend to go unnoticed, into public view.

The ways in which the profession takes an active role in emotion management during euthanasia is also demonstrated through Morris's (9) observations of conversation, physical contact and intimacy during the veterinary encounter:

*Most of what we do in euthanasia with owners is not technically a part of our job as veterinarians. I know this stuff is above and beyond, but I feel that it* ***is*** *my job. It's like that is our part as part funeral director. It's not like we're just doctors. We're part healer, part grief counsellor, part funeral director, so of course we have got that feeling that [it] is our responsibility to make euthanasia go well for the animal and the owner (p. 100)*

I will later argue that such ethnographic detail on the contextual role and purpose of the vet may usefully challenge externally imposed ideals of veterinary professional boundaries.

Finally, Morris (9) observes how the novice veterinarian is uniquely challenged by euthanasia and that coping with this practice is a skill which must be learnt:

*After all these experiences, I often thought, “Are these vets made of steel?” Trained and committed to saving the lives of animals, how do they resist adopting all those animals they would rather not euthanize? Whether a pet faces a life-threatening illness or needs an allergy shot, veterinarians have to be prepared to assume the appropriate mood required by the job. They walk into euthanasia scenes, focus on the animal, stroke their fur, administer the injection, and say to the grieving client, “She's gone”. How do they go from that scene directly to the next room and smile at the puppy with a lacerated paw? How do they not cry when someone leans over her dog or cat as he is dying and says, “Yes, yes darling, you are a good boy. Everything is going to be alright. You are such a brave boy. Mommy loves you.” Many times I had to ask myself, “How do they do it?” The answer is, in part, that in the beginning they did not. There are a lot of veterinarians with one-eyed, three legged, diabetic pets who had been slated for euthanasia. Many veterinarians shed tears during the euthanasia procedures, while others tucked themselves away in a bathroom only to emerge red eyed from crying (p. 109)*

Whist moral stress has become a topic of increasing prominence within the veterinary literature (10), the detail of the emotional challenges involved in veterinary work, as highlighted through ethnographic study, deserve far greater attention. I will later argue that the desensitization of vets through training and experience has significance for the way we understand veterinary ethics.

In summary, though exploring the detail provided by ethnographic studies from the fields of medical and veterinary anthropology, I have sought to show the potential of such research methods for bringing onto view new material, sensory, emotional, and practical understandings of the veterinary profession. As a vet I know that these aspects of the role are important. I will show though my own auto ethnographic account that they form a large part of my memories of a case I struggled with over 20 years ago. In the next section, I will make the case that such accounts of veterinary medicine are also *ethically* important, when viewed through the lens of a feminist ethical analytic approach.

### Feminist Ethical Analysis

In this section, I introduce the underpinnings and focus of feminist approaches to science, the social sciences and ethics in particular. Through a scoping review of existing literature in veterinary ethics and feminist ethics more broadly, I highlight the ways in which feminist care approaches to ethical analysis might develop the field of veterinary ethics.

#### Feminist Research Approaches

In both scientific research, and the social sciences more specifically, feminist approaches have been directed at the elimination of unconscious sexist bias in research. The feminist turn in science, as observed in the 1970's, was initially described as provoking gender based resistance:

*However, despite that zeal and promise, it still provokes resistance. Although surliness has historically been a common response to any new, invigorating intellectual stimulus, the new scholarship about women does inspire special forms of dislike, which are interestingly analogous to the response to women in society at large* [(11). P. 1]

The impact of feminist thought on the discipline of science was more clearly articulated and appreciated by the late c20. Challenging the notion of neutrality in science and embracing neurodiversity are two foundational impacts on the philosophy of science which have been attributed to women's influence on the field:

*Is maximizing the objectivity of research always advanced by maximizing the social neutrality of research processes? The neutrality ideal is maximally effective when it is invoked in contexts where social beliefs differ among members of the scientific community. But how is it useful in detecting social assumptions shared by an entire scientific community?*

*In a related way, attention to women's concerns has helped to reveal the value of cognitive diversity in the scientific process. Just as biodiversity is invaluable for human well-being (as well as a good in its own right), so, too, is cognitive diversity. […] “The” scientific method can be enhanced by our appreciation of the wealth of intellectual resources to be gained by valuing and promoting cognitive diversity* [(12) p.1599].

From a sociological, rather than scientific perspective, the feminist “standpoint” position has long argued that women, as a traditionally subordinated group, are in a different position when arriving at an adequate representation of social reality than men, and that women's everyday experiences of bodily work and intimate relationships can contribute to a truer picture of reality than that provided only by a masculinist science (13).

More specifically, in terms of method, feminist approaches represent the replacement of “objective” structured interviews and quantitative analysis by more reflexive and interactive unstructured data collection and a method of writing that is said to allow the subjects to speak for themselves. In terms of epistemology, one view is that striving after objectivity, truth and control over nature is a masculine urge; women are suggested to make less of a distinction between knower and known, self and other, mind and body, subject and object, and to be more tolerant of ambiguity and multiple truths (4).

Thus feminist approaches, whilst having their foundations in the observation of gender differences, have become well-developed toward challenges of traditional political, scientific and social systems which have a much broader contemporary resonance.

With regards to feminist influences on understandings of ethics, in particular, foundational feminist literature explored differences in psychological development and understandings of moral behavior between women and men (14). Through bringing women's voices into a previously masculine debate, Gilligan (14) identifies an alternate view of moral problems whereby they appear to arise through competing responsibilities, rather than competing rights. This, argues Gilligan, ‘*requires for its resolution, a mode of thinking that is contextual and narrative, rather than formal and abstract’* (p. 66). Gilligan's influential work develops images of relationship and self-identity to show that ‘*in the different voices of women lies the truth of an ethic of care, the tie between relationship and responsibility and the origins of aggression in the failure of connection’* (p.173). An ethic of care, it is argued, contrasts with an ethic of justice which proceeds from the premise of equality, resting instead on a premise of non-violence—that no-one should be hurt (p. 174).

Whilst the binary view of gender that much of this foundational work is based upon is fortunately becoming outdated, the origins of feminist care ethics might simply be described as the drawing of attention to caring relationships as fundamental in the definition of our moral obligations. Traditional justice based ethics, it is argued, are potentially aggressive through their failure to connect caring relationships with responsibilities, permitting abstract moral reasoning which does not account for context, emotions and vulnerabilities.

Early feminist approaches to bioethics applied ethics of care to biomedical issues which affect the genders differently, aiming at procreative policies which permit women to ‘*control their interconnected reproductive and genetic destinies’* [(15); p.98]. Again, in moving beyond gender categorisations, an initial focus on the ways that vulnerability and power inequalities traditionally disadvantaged women and children in healthcare settings has evolved in contemporary feminist bioethics to considerations of broader social and cultural influences within healthcare such as gender, disability, race and language (16). It is unsurprising to me, as a veterinary surgeon, that feminist interests in protecting the vulnerable and socially disadvantaged has led to work which is focused on ethical relations with non-human animals in our society (17). Whilst feminist explorations of biomedicine (18) have already engaged with non-human animals through examining their role within the laboratory setting, the place of animals as subjects and objects within veterinary medicine has yet to be explored in detail from a feminist perspective. Through bridging an opening which exists between feminist approaches in animal ethics and bioethics, this paper argues that feminist approaches to veterinary ethics might not be as distant as they currently appear.

#### Feminist Veterinary Ethics?

Here, I describe the relative youth of the field of veterinary ethics and the traditional ethical approaches upon which it has largely been based. I show how the critique of traditional reductionist philosophy is supported by the ethnographic studies of veterinary practice which I have explored. Finally, I show some of the ways in which feminist ethical approaches may develop veterinary ethical thinking.

Woods (19) suggests that in the late twentieth Century “*veterinarians began to recognise the potential conflicts in interest between the animal, owner, society and profession”* (p. 13). It is likely that this new perspective mobilized the development of the field of veterinary ethics, described even quite recently as embryonic as a dedicated field (20). In a similar pattern to that observed in ethical disciplines more generally, traditional reductionist philosophy has been very influential in veterinary ethical analysis to date, supported by the foundational works of prominent philosophers such as Regan (21) and Singer (22). In their broadest sense, the dominant deontological and utilitarian ethical theories promote either the rights of individuals or collective well-being as the purpose of moral behavior. In veterinary ethics this has been translated into analytic approaches which tend to both highlight and prioritize the needs of some individuals over others, or which aim to balance the needs of the majority and the significance of those needs.

Importantly, an accepted limitation of both these major theories, and some other less familiar ethical approaches, is that they assume impartiality in ethical decision making, whereby adherence to strict rules or numerical calculations can determine a morally correct course of action, in every setting. In veterinary ethics literature, it has been acknowledged that:

*Such an approach fails to acknowledge the reality of human relationships. Some argue that the more universal, abstract, impartial and rational ethical decision making is, the further it is from reality* [(23); p. 52]

Mullan and Fawcett (23) further point out that through stripping emotion from veterinary ethical decision making, these theories also risk ignoring salient aspects of a scenario, for example, that our emotions can make us sensitive to particular situations and inform our perception. Our emotions are also important, they argue, for empathetic responses to the needs of another (p. 53).

How important are these deficiencies in current veterinary ethical approaches? From a practical point of view, in the UK the veterinary profession's regulatory body The Royal College of Veterinary Surgeons (RCVS) acknowledges the potential for conflict in veterinary decision making, maintaining that vets should balance their professional responsibilities toward animals, clients, the profession and society, having regard first to animal welfare (24). That such a balance may be difficult to achieve has been highlighted most convincingly through recent work which examines the prevalence and significance of moral stress within the profession. For example, following a survey study of North American veterinary surgeons, Moses et al. (25) show that for 78% of respondents, not being able to do the right thing for a patient caused veterinary staff moderate to severe distress *(p. 2120)*. Moses et al. (25) tested the hypothesis that veterinary surgeons frequently encounter ethical conflicts which cause moral distress, but rarely recognize these situations as ethical or moral in nature:

*The implicit assumption is that veterinarians may not consider commonly felt distress as being triggered by a conflict between their actions and their personal morals. Instead, they may perceive the situation as “sad” or “upsetting” without acknowledging why (p. 2116)*

The authors argue that “*many veterinarians are distressed and anxious about their work and are troubled by many of the requests that are made of them (p.2121)”* and suggest that “*Recognizing, acknowledging, and labeling conflict and distress as ethical in nature are important first steps in combating moral distress (p. 2121)”*. That veterinary surgeons often feel distressed and anxious and that these feelings have an ethical basis supports this paper's overall argument for developing a more robust connection between lived experiences, veterinary storytelling and veterinary ethics.

Quain et al. (10) share thematic analysis of free text responses arising from a mixed methods survey of ethically challenging situations encountered by veterinary team members during the Covid-19 pandemic. Whilst not in ethnographic detail, this allowed participants to offer a personal glimpse into the realities of veterinary ethical conflict during lockdown. For example “*Home schooling an 8-year-old while working full time! How to deal with (euthanasia) home visits which I consider ethically essential.”* (p. 8). That family, society, employment and animal welfare might collide in veterinary ethical decision making illustrates the significance of understanding the *relationships* which define the multiple aspects of veterinary life. As Quain et al. (10) point out:

*Understanding the bases of these conflicts is critical in communicating about and potentially resolving them (p. 20)*

Whilst Quain et al. (10) identify the use of both rights and utilitarian ethical reasoning by veterinary surgeons through their data (p. 5), I argue here that this limited theoretical approach to veterinary ethics, as it is widely understood, does not help us to fully understand and communicate the ethical conflicts which form part of lived reality for the veterinary profession. I suggest that this is because it does not usefully engage with the relationships, emotions and sensory experiences which are part of the lived experience of veterinary decision making, as highlighted through the existing anthropological research I have explored above.

Whilst there is little, if any, literature which explores a feminist approach to veterinary ethics specifically, feminist approaches to animal ethics more generally do appear to engage usefully with these concepts. For example, Gruen (26) highlights that the many care traditions in animal ethics recognize that reason cannot be isolated from embodied experiences. Through their own analysis of human-animal relations, Gruen promotes an ethical practice which connects empathetic feelings between humans and animals:

*Entangled empathy is a process that involves integrating a range of thoughts and feelings to try to get an accurate take on the situation of another and figure out what, if anything, we are called upon to do* (26 p. 81)

That an ethical approach to animals might commence with thoughts and feelings, rather than be supported primarily by abstract ethical theory, is in stark contrast with current approaches in veterinary ethics.

Similarly, Engster (27) claims in their analysis of care ethics and animal welfare that it is the extent of an animal's dependency on humans which dictates our moral obligations toward them. They suggest that a care ethics approach to the moral treatment of animals is unique in that it grounds our moral duties to animals in our sympathy for, and relationships with them (27). Again, the elevation of emotions such as sympathy to a critical moral significance and the embedding of relationships within ethical decision making is unique and distinct from dominant ethical approaches in the veterinary literature. This view of the nature of human animal relationships in terms of dependency and vulnerability connects care approaches to animals with more developed areas of feminist theory, visible across the field more broadly.

In summary, I have here made the point that whilst feminist approaches to veterinary ethics are not well-developed, existing feminist care approaches to animal ethics attend to many of the acknowledged deficiencies in rights and utilitarian theory which are outlined in the veterinary ethics literature. A feminist approach to animal ethics more broadly has been show to engage with concepts which are identified in existing ethnographic accounts of veterinary encounters, such as relationships, emotions and empathy. A feminist care approach, unlike traditional ethical approaches, maintains that these concepts have a critical moral significance.

#### Feminist Ethics and Anthropology

Finally, I wish to highlight the philosophical and methodological synergies between ethnographic research and feminist ethical approaches. I argue that the value of veterinary anthropology for advancing veterinary ethics is through formalizing an engagement with relationships, emotions and concepts which cannot be reduced to abstractions, within the lived reality of veterinary practice. That these concepts have moral significance is a critical component of a feminist care ethic.

It is not necessarily the lack of anthropological studies of veterinary practice which have permitted abstract theories to dominate in the development veterinary ethics. Rather the reverse may perhaps be true, the acceptance of ethical approaches which do not require analysis in the real world may have delayed the development of detailed empirical studies of the profession. Indeed, the academic relationship between empirical studies of how our world *is* and normative (ethical) analyses of how our world *ought to be* is a highly contested and mobile area. In veterinary ethics and bioethics more broadly, the traditional philosophical roots of ethical theory imply that human activity should be a consequence of ethical deliberation, rather than vice versa. That this ideal is now challenged by those who argue that ethical deliberation is *in itself* a human activity supports the recent emergence of the somewhat contested middle ground of empirical bioethics (28).

Even here, the relationship between empirical and ethical is unsettled and the existence of a meaningful distinction between facts and values (29) is generally maintained, even where the two might be argued to be symbiotically related (30). In veterinary ethics, empirical data has only very recently played a visible role, for example, survey data has been used to support analysis of the profession's ethical beliefs (31). In my own work I have argued that qualitative analysis of decision making in the veterinary setting gives rise to novel ethical questions for both the veterinary profession and for human medicine (32). Whilst such work conforms to the careful bioethical understandings of empirical ethics, a relationship between anthropological work and feminist approaches might more radically refocus veterinary ethical thinking.

One key aspect of feminist care approaches is the altered relationship between conceptual and practical ideas of ethics, compared with traditional bioethical approaches. At the beginning of the section Feminist Research Approaches, I described the trend toward interactive and unstructured research methods, which is characteristic of contemporary feminist research approaches. This represents a further indication of the significance of lived reality, over predetermined structure, within feminist care approaches. How then, does such an approach affect the already contested relationship between factual and moral argumentation? In my view, feminist care approaches further challenge both the rationale and capacity for the separation of factual and moral reasoning. This presents an undoubted challenge to the current supremacy of isolated moral codes, particularly within scientific and political systems, whereby philosophical determinations of ethical activity may not match up to those being evaluated at an individual level.

Finally, as one example of the potential for a radical resetting of ethical thinking, Latimer and Puig De La Bellacasa (18) propose a duplicitous meaning of “ethics” exists in the laboratory setting. They define Ethics with a capital E as ‘*a fixed and vertically experienced, normative domain’* (p.159), this being the motivation of institutionalized approaches to ethics, which might also feel familiar with regards to current veterinary ethical approaches. In addition, however, they describe the everyday ethics, with a small e, which is brought into view through the trope of care. They argue that the ways that something is done may tell us more about the possibilities of ethics in practice than the normative ethical grids which seem to be controlling the field.

## Results

In this section, I demonstrate the use of ethnographic and feminist care approaches together, for developing our ethical understanding of veterinary medicine. First I share a novel auto ethnographic veterinary story, followed by a feminist ethical analysis of my own experiences. Both are then discussed for their relevance to veterinary practice and for developing a veterinary ethics of care in the final section of the paper.

### A Veterinary Story

*I can still see the dog's face as its eyes connected with mine, framed by the black bin bag it had been carried in. I can still hear the clicking sound, louder than the animal's desperate cries, made by a mass of maggots moving against one another beneath the dog's matted fur, crusted with faeces and fluids leaking from its damaged flesh. My hands were shaking with panic and rage and I could hardly draw up the lethal blue liquid into the syringe quickly enough. I wanted to put an end to this, immediately. As the needle pierced the skin I drew back a spurt of blood and slowly depressed the plunger, the fluid flowed into the tiny vein and the dog's body finally relaxed, as did I. At my hand, like so many others, she had ceased to exist, I have never felt so certain that I had done the right thing. Over the gathering silence only the sound of the maggots remained, the nurse's tears splashed onto the black plastic as she gathered up the bin bag and carried out their mobile feast. Through the window I could see the animal's owners waiting outside in the sunshine to pay me*.

*Fury propelled me into the car park, but I was instantly deflated by the woman's sobs as she smoked furiously and cried that she had no idea the dog was so unwell. She told me the dog had lived in a shed and they did not know about the diarrhoea, or that she could not clean herself properly. The maggots had set in so quickly, she insisted, that before they realised anything was wrong it had been too late. The man shifted uncomfortably and looked at his feet next to the still open boot of their car, he did not want to be here and yet, they had not left. They had not wanted to see or touch their own dog as they had rolled it into a bin bag to protect their car from its ruined flesh. They had not wanted to be with it when it died. These people were relieved and ashamed but they were also upset. I thought about the silky feel of the fur which had covered an expensively shaped head, I knew this dog was loved once. As the man took his wallet into the reception area the woman wearily closed the boot and got into the car. The process of paying a modest fee for euthanasia seemed to draw a partial line under their grief and guilt. Whilst I could not look at them as they drove away, they thanked me for ending the dog's suffering, and their own*.

*I returned to the waiting room where an RSPCA Inspector was sitting with a cat box on the worn vinyl floor beside her legs. Inside, a young healthy cat purred loudly and pressed its face against the plastic mesh. The Inspector smiled warmly at me when I came into the room and the sun coming in through the window behind lit up her hair. My frozen face refused to respond to her friendly smile, which faltered as she took in my defeated expression. I needed to check the kitten had healed after a routine neutering operation, but a rushing sound had filled my ears and adrenaline fogged my vision as I tried in vain to refocus on my next appointment. I felt as if I was dragging the weight of that poor dead dog into the waiting room behind me, leaving a trail of maggots in my wake*.

*In the end it was a relief to share the burden of what had just happened. The black plastic bag was once more peeled back to reveal the smell of putrid flesh and the maggots still hard at work on the rear end of the deceased dog. Above the cheerful purring of the kitten in the cat box, now balanced on the edge of the countertop, I felt comforted by the whirring of a camera shutter as the inspector evidenced the dog's suffering. Now it was not just my problem to carry, someone else cared*.

‘*It's up to you of course’ said the senior vet when I told him what had happened later that day. He was standing in the centre of the busy preparation area, rocking back on his heels and looking at the ceiling as he thought about what to say to me. ‘But I would be careful about involving the RSPCA in a case like this, because it could mean that next time the owner might not bring the dog to the vet at all’. Of course, I did not think that he literally meant the same people might appear again in the future with another dog in a similar state, at least I hope he didn't. What I think he meant was that vets need to be available, without judgement, to do what is right at the time for an animal who is suffering. I was aware of the discreet glances of the veterinary nurses who could overhear our conversation, whilst folding clean operating drapes and scrubbing instruments. It was not the first time in my short period of employment that he had disagreed with my actions. His words troubled me as my fury and righteousness abated over the following days and I began to lose confidence in my decision*.

*When the time came to speak to the Inspector again I took the call in the under stairs cupboard. Stretching the phone cord through the door hinges, I shut myself away at the top of the cellar steps. The cellar held a dusty collection of the more private aspects of veterinary practice, body bags and calving jacks and quite fittingly the chest freezer, which held the remains of whole animals and redundant body parts until they were collected for cremation. The cold, dark setting perfectly fitted my mood as I explained that I was not going to support a prosecution for animal cruelty after all. Of all the upsetting and stressful calls I have made as a vet, giving bad news and arguing over bills or apologising for the ineffectiveness of my treatments, this was the only one of which I was ashamed. I am fairly certain the nurses and receptionist could hear my voice through the flimsy cellar door, but at least I could only imagine the look on their faces. The lovely kind Inspector who had helped me through that terrible afternoon listened quietly as I repeated the reason and she had the good grace not to try to change my mind. The disappointment and resignation in her voice were almost, but not quite, as unbearable as her obvious lack of surprise*.

Veterinary stories like this are not easy to find in veterinary journals. I can tell this story because I am a veterinary surgeon, but it is published here as an example of how the emerging field of veterinary anthropology may provide an important new perspective on veterinary practice.

Other than the humorous tales of James Herriot (33) and heroic stories of The Supervet, (34) who tells the story of veterinary medicine? Who connects the experiences of animals, owners and vets and the tensions between them in the detail that we experience in practice? It is here that veterinary anthropology will make a difference.

In the next section, I will use the work of feminist theorist Donna Haraway (35) to connect ideas of storytelling and ethics. Here, I share her perspective that “*It matters what stories we tell to tell other stories with”* (p.12). To me, it matters what stories we tell of vets, to tell the story of the veterinary profession.

### A Feminist Analysis of Veterinary Lived Experience

In my story above, I describe my feelings of relief that I had ended the dog's suffering and my absolute certainty that I had done the right thing by ending its life. I knew that she was too unwell to recover and was suffering to such a great extent that immediate euthanasia was my only option. I did the only thing that I could do as a veterinary surgeon, for an animal under my care. I am satisfied that my actions met my professional obligation to have regard first to animal welfare (24).

However, the story also tells how this case ultimately left me feeling confused and uncertain about where the boundaries of my professional responsibility lay. Whilst I had absolute certainty about what I should do for the animal itself, I was very much less certain about my handling of the surrounding situation. What else should I have done? How could I prevent this happening again? Why did I feel so unhappy?

In this section, I analyse the story using a feminist perspective. As outlined in the section on methods above, such an approach differs markedly from traditional theoretical analytic techniques, both philosophically and methodologically. Whilst a feminist analytic approach to veterinary ethics has yet to be described, here, I draw on key concepts identified through existing feminist literature and apply them to the novel auto ethnographic data I have presented above. In acknowledgment of both the novelty and scope of change which such an approach represents in veterinary ethics, I aim only at this stage to show how engaging with such ideas might broaden our ideas of what veterinary ethics is and what it does. In Matters of Care Puig de la Bellacasa (36) asserts a similar stance, suggesting that “*I resist categorising care but rather seek to emphasize its potential to disrupt the status quo and to unhinge some of the moral rigidities of ethical questioning [p*. *(**11**)]”*. In the final discussion section I explore what such a radical reframing of veterinary ethical analysis might mean for the profession.

#### The Feeling of Care and Emotional Juxtaposition

Through exploring care as a speculative ethic for more-than human worlds, Puig de la Bellacasa (36) asks ‘*what does caring mean when we go about thinking and living independently with beings other than human, in “more than human” worlds? (p. 13)*. The author emphasizes that ‘*a politics of care engages with much more than a moral stance; it involves affective, ethical and hands-on agencies of practical and material consequence' (p. 4)*. An ethic of care, at its most basic level, identifies caring as a moral imperative. How care is done, what care is and is not and who should care for whom become critical questions within the development of such an approach. When thinking about the complexities of defining “care” in these terms, it becomes clear that there is much work still to be done here, both in the veterinary field and elsewhere. As a starting point, however, one critical aspect of care ethics, which sets it apart from more traditional ethical approaches is the observation that an ethic of care may permit us to start with, rather than end our ethical analyses with reference to our feelings (26).

What is unusual then, for veterinary ethical analysis, is that engaging with a feminist ethic of care permits us to consider what ethics *feels* like, as well as what it does. What happens to our ethical perspectives when, unlike traditional ethical analyses, feelings take center stage in a feminist analysis?

Many negative feelings are described through my story; repulsion at the sight, sound and smell of a mass of maggots; sadness at the death of the dog; fury at the owners waiting in the car park to pay; their distress, relief, shame, grief, guilt and gratitude; the nurses' disapproval of my colleague; my own frustration and shame and the disappointment and resignation of the inspector. From a feminist perspective at its simplest, this emotional analysis of a veterinary encounter tells us something important about the event, it tells us of its moral complexity.

If we think about the different ways this story might be analyzed, of the generalizations that an analysis using rights based reasoning could be based upon, or the simple facts that a utilitarian calculation would require, we see what the “stripping of emotion” (23) has taken away from veterinary ethics. In the search for a simple overarching solution to all ethical challenges, each personal struggle, every hard won fight and the frequent emotional pain of attempting to achieve what is right is removed from the analysis.

If ethical decision making is required to be logical and rational, emotionally complex experiences of being a vet do not *feel* like an ethical problem, purely because they do not *feel* logical, they do not *feel* rational. This particular case *felt* impossible to resolve, but there is no room for ethical impossibility in traditional veterinary ethical approaches, which assume that there is always at least one justifiable course of action. Might a feminist approach suggest that when an ethical outcome *feels* impossible, it signifies something important, such as a level of ethical complexity or disagreement which requires further unpacking?

Moses et al. (25) have already identified a disconnection between veterinary emotional states and vets' identification of ethical conflicts. It might be argued that the common veterinary understandings of ethics as distinct from emotion may be unhelpfully restricting the application of ethical reasoning in practice emotional reactions must signify what is important to us personally, might they not be considered as intrinsically of ethical importance when considering how we should act, regardless of the ethical framework used?

In fact, the emotional complexities of this case do not end with the variety of negative feelings it aroused. In my story I also refer to many positive feelings; love, compassion, friendship and care. I also describe the jarring impact of the sunshine and the purring cat and the way that I felt comforted by the whirring of the inspector's camera shutter and what it represented. A feminist care analysis which attends to all the different emotional states in this veterinary story highlights that it may be the juxtaposition of contrasting material and emotional states which *feels* the most difficult. Indeed, my memories are dominated by the crashing of gears in my mind as I attempted to put aside the dead dog, the maggots and the guilty owners whilst taking in a smiling face, a purring cat and the sunshine coming through the window. In the other anthropological accounts we have examined, such scenarios also arose for Jake as he cared for and killed the worms (7), and for Morris's euthanasia vets who had to walk next door and smile at the puppy (9). That different emotional states might coexist or collide invites, through a feminist analysis, notions of a complex layering of ethical states in the veterinary clinic.

Gruen (26) asserts that we need to be more honest when sharing the details of human-animal relationships. My story and those of all vets gives a view of our world which no other individual can see, making it the profession's responsibility to share our unique perspective, and to share it fully. A feminist analysis of this honest account of a veterinary encounter, centered on emotion, highlights that there is something particularly difficult about a vet's involvement in human-animal relationships which can often combine love, neglect, tenderness and violence. In order to understand this more fully we may choose to consider the uncomfortable possibility that these feelings matter, in an ethical sense. Rather than ignoring or supressing these emotions, a feminist approach might oblige us to explore whether they are alerting us to wider ethical problems in the veterinary profession and in society.

Through a feminist analysis centered on emotion I have developed an argument for the role of feminist approaches in helping us to better recognize and understand the complexity of veterinary ethical decision making. In the next section below, I will develop the argument that feminist approaches may also expand our understanding of the necessary scope of veterinary ethical debate.

#### Tentacular Thinking and Relational Structures in Veterinary Care

In this section, I analyse my story again, applying an influential feminist perspective to the nature of relationships in veterinary practice.

The work of multispecies feminist theorist Donna Haraway may be unfamiliar to most veterinary audiences. It is influential for those with an interest in reimagining relationships between humans and non-humans at this critical time in our shared history, which many of the profession may be. In Staying with the Trouble, Haraway (35) offers new ways to reconfigure our relations to the earth and all its inhabitants. The argument is made that relationships might be imagined as tentacular, comprising a complex web of connections, which extend outside only human interactions. Tentacular thinking represents a challenge to traditional understandings of human relationships, whereby they have been considered as distinct and separate from our interactions with non-humans in all their forms. This way of re-imagining relationships on earth is radical and in a feminist tradition, challenges the many ways that a masculinist society has distinguished itself, making artificial ‘*The purifying division of society and nature (p. 41)’*.

Tentacular thinking, I suggest, also offers a way to reimagine relationships in the veterinary setting, from a feminist perspective. The RCVS Code to professional conduct (24) highlights the multiple responsibilities which veterinary surgeons face, and these have been contemplated in terms of multiple veterinary relationships in recent veterinary ethical approaches (37). Through my story, I have identified my discreet interactions with the dog, my clients, my colleagues and the RSPCA inspector. That I am expected to balance my professional responsibilities to each (24) is problematic, in part, because it neglects the reality that they are also involved in complex interactions with one another. The multiple relationships which exist in the veterinary setting are constantly shifting and becoming reorganized. How can one effectively balance the components of something which is never settled and is never still?

For Haraway (35) ‘*Tentacularity is about life lived along lines- and such a wealth of lines- not at points, not in spheres’ (p. 32)*. In this view, we engage with a perspective that connects all the complexities of interactions in our lives in a linear sense. From a tentacular perspective, then, the dog in my story connects me to their owner, but the dog also connects me to the RSPCA inspector and, through me, the dog connects the RSPCA inspector to their owner. All the animals under the care of the practice connect me to my colleagues and, though them, we are all connected to society at large. Our professional guidance and the mode of thought applied in traditional veterinary ethical analysis observes only the ways in which we might usefully separate out and circumscribe our responsibilities (44). Through tentacular thinking we are charged with acknowledging the interconnectedness of them all. Instead of individualizing our interests, our world becomes a web of interactions and ‘*Whether we asked for it or not, the pattern is in our hands’ (p.34)*.

Haraway (35) suggests that we are all responsible for multispecies flourishing, though not in the same ways. In veterinary practice, the pattern of our tentacular web is created through our unique relationship with animals, conceptualizing relationships, responsibilities and dependencies which circulate around and become exposed *specifically* through veterinary interventions. Tentacular thinking finally offers a view of our profession which acknowledges the impossibility of separating out our professional responsibilities. Further, it accepts the pivotal role we play, not as freestanding independent arbitrators, but as *mediators* in the complex relationships between humans and animals, and all their far reaching implications.

Here, to show how radically tentacular thinking might alter our view of what veterinary ethics and ethical activity is, I turn to Morris's (9) reminder that veterinary euthanasia is a relatively recent phenomenon and that before the mid-twentieth century, animal owners treated, nursed and even killed their animals at home (p. 3). Tentacular thinking shows us that by assuming the practical and emotional task of ending the lives of animals, vets inevitably alter the lives of those and other animals, their owners and society more broadly. These ongoing alterations will, of course, be beneficial for some individuals, but for others they will not. Tentacular thinking causes us to ask what else might have changed, and for whom, if I had not euthnased the dog?

Such radical thought is likely to be unsettling, Haraway herself contemplates the value of stories where ‘*there are only more and more openings and no bottom lines?’ (p. 29)*. Such an approach will remain unappealing for those who require an ethic that responds only to a call for black and white decision making. However, tentacular thinking illuminates the very real risk that traditional approaches may cause us to only observe and aim to resolve one part of what are much more complex ethical problems.

#### Emotional Sponge Work

In this section I propose the concept of “emotional sponge work” as potentially helpful for developing understanding of the societal role of the veterinary profession from a feminist perspective.

Through describing a feminist perspective on emotion in my analysis of the story above, I have sought to show how difficult it is for vets to respond emotionally, or to not respond emotionally, amid the continuous reorganization of relationships, sensations and materials which occurs within the veterinary clinic. Through contemplating the movement of emotions themselves within the veterinary clinic, I now raise the perspective that my own emotions in relation to the story told above may represent the crucial role I played in appeasing the grief and guilt of the dog's owners. In the same way that I describe how I benefitted when my burden of care was shared with the RSPCA inspector, the intense emotions associated with the dog's suffering might also be described as having been transferred, at least in part, from the animal's owners to me. After the dog's owners had left the clinic, feeling slightly better, the worry of what to do about this tragic case had become my problem and no longer theirs.

Recent approaches in psychology have informally used the phrase “emotional sponge” to describe sensitive personality types which may become burdened by others' emotions (38). The phrase has also been used to describe complex professionalized emotional labor, as undertaken in education settings by peer writing tutors (39). Both through my story and the published ethnographies I have explored, I suggest that we can see examples of where the veterinary profession appears to function as an emotional sponge, with regards to society's relationships with animals. Through kindness, professionalism and obligation vets appear to gather, supress and tidy away the messy human feelings which come into the clinic, alongside their animal patients. Vets must not only hide their responses to these negative emotions within their own professional personas, they must also hand out sympathy, compassion and other appropriate emotions, when required.

Aside from the toll that I believe emotional sponge work takes on veterinary teams, I suggest that this perspective highlights one clear reason *why* veterinary emotions have ethical significance. Through cleansing society of the obligation to feel the unfiltered consequences of their relationships with animals must mean that the profession facilitates, to some degree, the emotional maintenance of human-animal relationships in their current form. The idea that veterinary emotions play a functioning role in society's treatment of animals both supports and is supported by a feminist approach to veterinary ethics. Gruen (26) points out that we must give moral attention to “*complex relations which involve more than suffering*” (p. 37).

The emotional sponge theory also has implications for the ethical development of the profession itself. Morris (9) shows us how vets become desensitized to the emotional intensity of their own work through the process of “learning” veterinary professionalism. In the same way that society's activities may be influenced by the alleviation of associated emotional burdens, what impact has “learning” to suppress emotions associated with veterinary work had on the development of veterinary professional norms? Furthermore, if veterinary professionalism is learnt through exposure to the norms of the profession, from where did these professional norms originate?

Using the concept of emotional sponge work I have shown how a veterinary ethic of care may require us not only to attend to the emotional states of veterinary professionals, but also to distinguish them from the learnt responses which the profession requires. In this way we see how anthropology has the potential to do far more than inform our ideas of what veterinary professionalism *is*. Through an increased attention to the affective states of vets, an ethic of care approach urges us instead to alter our ideas of what veterinary professionalism *should* be.

In the final discussion section below, I explore the wider implications of these feminist analyses for the field of veterinary ethics and the veterinary profession.

## Discussion: Toward A Feminist Ethic of Care for the Veterinary Profession?

Through the feminist analyses of veterinary practice above, I have identified the considerable scope and scale of changes to current understandings of veterinary ethics which a feminist approach might bring. In this final discussion section, I explore what developing an ethic of care for the veterinary profession might ultimately achieve, both for the profession and the field of veterinary ethics.

Critiques of feminist care approaches from within the profession suggest that it is confusing, vague and underdeveloped (23). My own view is that such perspectives arise only when feminist approaches are viewed through the lens of a traditional reductionist philosophy, which has embedded within veterinary ethics the language of rules, calculations and impartiality. From a different perspective, and one which I hope I have portrayed, feminist care approaches are potentially rich, detailed and expansive. Gruen (26) reminds us that attention is a central feature of care ethics (p. 35) and I would suggest that the observed confusion and vagueness arises from our previous lack of attention to the aspects of veterinary practice which might inform a care approach. In my analyses above I have shown how the concepts of relationships and emotions, which are not attended to in traditional ethical analyses, may become better understood through developing a feminist veterinary ethics.

I do, of course, concede that a feminist veterinary ethics approach is currently underdeveloped, this paper aims to begin the conversation about how we might develop such an approach. I hope that I have also shown that veterinary anthropology and ethnographic work will help us on that journey through illuminating aspects of veterinary professionalism which we have not previously been attended to, such as emotions and relationships in veterinary practice.

Current professional guidance asserts that vets must make animal welfare their first priority (24), and traditional veterinary ethical analyses have been carefully applied to advancing this agenda by many, including myself. Following my story told above, however, I was keen to point out that my actions in euthanizing the dog *did* attend to her welfare, but that was not the end of the problem. Whilst I share this veterinary ideology, the problem in reality is, and always has been, that separating animal welfare from all the complexities of our professional relationships is rarely possible (40). The feminist analyses presented here challenge the boundaries of veterinary ethical thinking as they are currently understood, including the current limitations of veterinary ethical concern. Tentacular thinking has helped to show that separating animal welfare from other concerns risks shifting our focus away from ethical problems elsewhere, which might actually underpin the problem. Donovan et al. (17) reminds us that attention to individual animal suffering, or an animal welfare approach, is not the same as attending to the political and economic systems which are causing the suffering, an ethic of care approach “*insists that these causal systems be addressed (p. 3)*”.

The profession's existing and valuable work toward improving society's treatment of animals must be acknowledged (41). However, we may still have cause to honestly contemplate whether the profession's stewardship of animal health could have been used to perpetuate unhealthy relationships between humans and animals (42). A veterinary feminist care approach might provide a framework for redefining the focus of veterinary professional responsibility, beyond the health of animals and toward the maintenance of healthy relationships between humans and animals. Of course, what a healthy relationship is then becomes a point of concern; a feminist approach to how caring responsibilities might be carried out and even politicized (43) might be thought of as ongoing and cyclical, whereby our developing understanding of care both informs and evaluates the health of our relationships.

A veterinary ethic of care might highlight that we are currently focusing too heavily on the detail of individual clinical decisions without acknowledging the unhealthy relationships which can often surround them. An ethic of care might lead us to question the degree to which ethical practices can ever be defined solely within the limited space of the veterinary encounter. Importantly, this approach would allow us to explore how the field of veterinary ethics connects with the disciplines defined as animal ethics, environmental ethics, food ethics and medical ethics, since we would acknowledge the impossibility of isolating the veterinary encounter from all the complexity of societal relationships with animals.

Whilst such a reframing of veterinary ethics appears daunting in its scale, I wish to draw attention to one reason why advancing such an approach may be incredibly important. I very much hope that through ‘*embracing vulnerability with purpose’* (5), the honest telling of my own story has been worthwhile. The concept of veterinary moral stress is a very real and very complex problem. Acknowledging that current veterinary ethical approaches are not sufficient could be critical, at the very least, for helping us to properly understand and develop meaningful approaches to veterinary moral stress.

In closing, as a scientist, I can understand how veterinary science has avoided interacting with ethnographic methods, along with relational ethical concepts and theories. However, as a clinician, what I know of veterinary practice and the fears and hopes I hold for the profession cannot be told using only scientific methods and language, nor adequately analyzed using only rules and numerical calculations. Finally, as a researcher, the emergence of veterinary anthropology as a discrete sub discipline is important, because it liberates a perspective on the profession which has been effectively hidden from view. I suggest that a feminist care approach becomes a logical and necessary addition to veterinary ethical analytic techniques, once such perspectives have come formally into focus.

## Data Availability Statement

The original contributions presented in the study are included in the article/supplementary material, further inquiries can be directed to the corresponding author/s.

## Ethics Statement

This study does involve human participants, however, the requirement for full ethical review has been waived by the University of York, cross-faculty ELMPS ethics committee. The grounds for this decision are that the author is the only participant in this study and the data is based entirely on the author's own past reflections and experiences (that is the data is based entirely on autoethnographic research). All references to individuals encountered during the period of autoethnography which are applied in this article have been fully anonymized.

## Author Contributions

The author confirms being the sole contributor of this work and has approved it for publication.

## Funding

This work was supported by the Wellcome Trust [219804/Z/19/Z].

## Conflict of Interest

The author declares that the research was conducted in the absence of any commercial or financial relationships that could be construed as a potential conflict of interest.

## Publisher's Note

All claims expressed in this article are solely those of the authors and do not necessarily represent those of their affiliated organizations, or those of the publisher, the editors and the reviewers. Any product that may be evaluated in this article, or claim that may be made by its manufacturer, is not guaranteed or endorsed by the publisher.
